# An Intensive and Comprehensive Aphasia Program Versus a Conventional Speech and Language Therapy: A Dose‐Controlled, Crossover Experimental Study

**DOI:** 10.1155/srat/2669488

**Published:** 2026-06-13

**Authors:** Cherie Wan-Yin Wong, Anthony Pak-Hin Kong, Ada Wai-Sze Chu

**Affiliations:** ^1^ Department of Human Communication, Learning and Development, The University of Hong Kong, Hong Kong, China, hku.hk

**Keywords:** aphasia, Cantonese, case report, ICAP, intervention, SLT

## Abstract

Aphasia, an acquired communication disorder after a stroke, negatively impacts an individual′s participation and communicative exchanges in daily life. Speech and language therapy (SLT) serves to either restore or compensate for language capabilities. An intensive and comprehensive aphasia program (ICAP) that emphasizes improving an individual′s abilities for daily participation and functional communication through a highly intensive schedule has accumulated substantial evidence supporting its efficacy. This study is aimed at comparing the effects of the mentioned treatment models to firstly progress the investigation of an ICAP in comparison with a conventional model, and secondly, at addressing local service gaps and identify how an ICAP could meet the needs of individuals with aphasia. A nonrandomized, dose‐controlled crossover, pre–post design. The ICAP and the conventional SLT (c‐SLT) were delivered alternately at a local community‐based rehabilitation center in Hong Kong, with a minimum of six‐month washout period between programs; treatment allocation was determined by the timing of recruitment. Twelve right‐handed adults with chronic aphasia were recruited. Both the treatment models provided 39 hours of treatment, using evidence‐based approaches and targeting both word and beyond‐word levels. The ICAP phase was delivered over 2.5 weeks at an intensity of 3 hours/day, 5 days/week. Treatment components included individual impairment‐based, participation‐based, technology‐based, and group therapy. The c‐SLT phase was delivered at an intensity of 2 hours/week, once weekly, providing individual language‐only therapy. Each participant received a total of 78 hours of treatment; no dropouts were recorded. Linguistic performance was assessed using two standardized comprehensive language‐functioning tests, an individual naming test and a discourse test. Communication confidence and effectiveness were also evaluated. Overall, the ICAP condition demonstrated better linguistic and quality of life treatment outcomes at the individual and group levels. Feedback on the experiences with the two treatment models was collected through interviews, which provided insights into perceived benefits from the user perspective and insights for future participant recruitment.

## 1. Introduction

Aphasia is a neurological condition characterized by impaired language abilities that can affect various aspects of communication, including speaking, understanding, reading, and/or writing, ranging from the lexical to the discourse level. Amid communication challenges, speech and language therapy (SLT) is often introduced to people with aphasia (PWA) to restore or compensate for impaired functions. Brady et al. [1] systematically reviewed 27 randomized controlled trials comparing SLT with no SLT and found that SLT significantly benefits PWA following a stroke, specifically by improving functional communication, reading, writing, and expressive language skills.

SLT varies in many ways, for example, in approach types (impairment‐based vs. functional‐based vs. caregiver‐focused), in modes of delivery (in‐person vs. virtual, or individual vs. group vs. app‐based), and in treatment intensity (intensive vs. distributed treatment schedule). A typical SLT model focuses on a single approach and is delivered at a relatively low intensity. Comparatively, an intensive and comprehensive aphasia program (ICAP) is an aphasia treatment model that provides highly intensive training to a cohort of patients, targeting both impairment and communication in daily activities and participation as defined by the WHO‐ICF [2]. An ICAP infuses evidence‐based practices to provide individual and group therapy interventions to facilitate the patient′s communication goals [3]. Over the past 2 decades, more than 25 ICAP studies have been reported [4]. A growing number of studies have identified the benefits of ICAPs, including improved receptive and expressive abilities [5, 6], functional communication [7], and psychosocial effects [8]. Recently, some ICAPs have been adapted for non‐English‐speaking populations, such as Brazilian Portuguese [9], Cantonese [10], and Swedish [11], demonstrating positive effects on linguistic skills and quality of life outcomes. Beyond performance‐based measures, Baliki et al. [12] demonstrated neural reorganization following an ICAP intervention using neuroimaging. Evidence supporting ICAPs is consistent with numerous systematic reviews [13, 14], which indicate that higher‐intensity treatment yields superior outcomes compared with lower‐intensity treatment. However, Brady et al. [13] also pointed out that the potential benefits of high‐intensity treatment may be confounded by a higher dropout rate. The ICAP study by Rodriguez et al. [5] reported higher dropout and fatigue rates in the 100‐hour ICAP experimental arm than in the 40‐hour arm, and the study by Wong et al. [15] reported a lower mean dosage delivered in the in‐person ICAP than the virtual one. However, the study by Dignam et al. [16] found a comparable attendance rate between the intensive and nonintensive groups. These observations raise an interesting question: whether the highly intensive model itself or personal factors are influencing the dropout rate or attendance.

SLT in Hong Kong is typically provided to patients with aphasia through (i) hospitals′ outpatient services, (ii) community‐based rehabilitation centers, or (iii) private clinics. The intensity, dosage, and types of treatments varied across settings, highly dependent on an organization′s resources and the speech therapist′s professional judgement. A stroke patient in the acute stage primarily received SLT focused on swallowing assessments and communication screening. During the subacute stage, such patients are frequently referred to rehabilitation hospitals and undergo regular therapy sessions. Upon discharge, a referral letter is provided for outpatient follow‐up, and patients are required to complete the Hospital Authority′s registration process to schedule appointments. Meanwhile, many families seek assistance from community resources, including nonprofit organizations, district health centers, or self‐financed sectors, to access SLT for their poststroke family members. Kong [17] reported that most inpatient and outpatient speech and language services for aphasia were delivered weekly and monthly, respectively, in fewer than 30 min in the early 2010s. Another survey study on local aphasia rehabilitation services, conducted a few years later [18], identified that the intensity and frequency of services were still inadequate, with aphasia treatments of once/week, 1–2 h/session at the university and private clinics being the closest to the recommended level. The frequency and intensity of aphasia service delivery were even lower in other settings, with nearly half of clinicians providing aphasia treatment once a month, and over 75% of outpatient services were around 30 min per session. The study also revealed a lack of comprehensive treatment in the local aphasia service, which was predominated by individual language therapy, followed by communication partner training interventions.

To better address gaps between research evidence and expand the knowledge base for managing aphasia in Chinese, we aimed to identify and compare the effects of an ICAP and conventional SLT (c‐SLT) in native PWA using a dose‐controlled, crossover experimental design, in which all participants were exposed to both treatment conditions. Additionally, interviews with participants were arranged to gather qualitative insights into patient experiences, which could enhance understanding of the impact of ICAPs in the healthcare system [19].

### 1.1. Aim

i. Identify the effects of an ICAP and a c‐SLT on linguistic abilities and quality of life outcome measures, at both the individual and group levels
ii. Compare the effects between the ICAP group and a c‐SLT group
iii. Explore how the participants perceived the two treatment models

## 2. Method

### 2.1. Design

The study used a nonrandomized dose‐controlled crossover design. This report followed the CARE guideline checklist and is available in Supporting Information 1. Four cohorts (six sub‐groups) of ICAPs and four c‐SLT programs were held at a local community‐based rehabilitation center in Hong Kong from July 2024 to December 2025.

**Figure 1:**
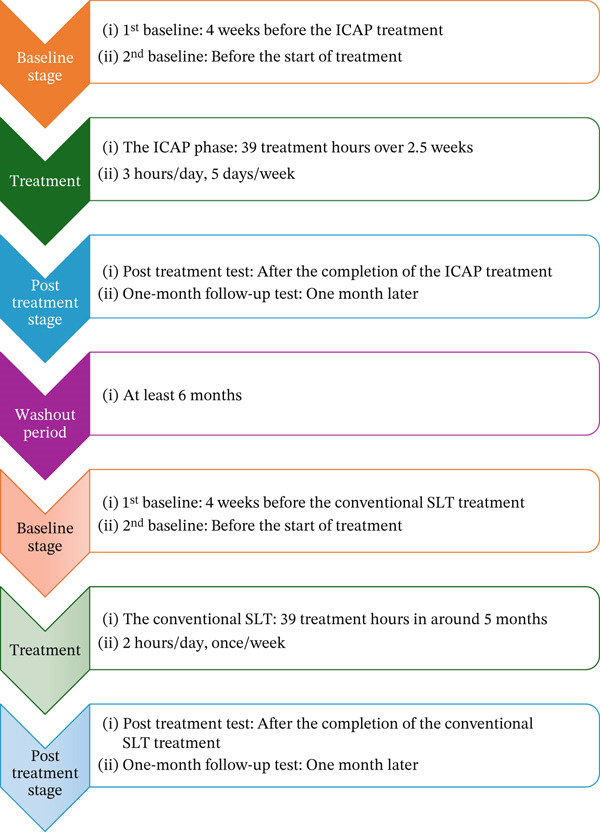
A demonstration of a participant who first received the ICAP intervention, followed by a washout period, and then received the c‐SLT intervention in this crossover study.

All eligible participants received both a 39‐hour ICAP and a 39‐hour c‐SLT program, with a minimum washout period of 6 months between programs. Given the nonrandomized allocation in this study, the order in the crossover design was tested as a covariate in the linear mixed model and found to be nonsignificant. The two programs were held alternately, with treatment allocation determined by the time of recruitment. Participant recruitment started by sharing the recruitment poster through the rehabilitation center′s network. Participants meeting the inclusion and exclusion criteria were recruited on a first‐come, first‐served basis. Although the recruitment process and procedures remained consistent throughout the study period, selection bias may arise because the willingness to participate in both treatment models may vary among eligible participants, resulting in a recruited sample that may not fully represent the population. Additionally, changes in personal or environmental factors during the treatment period may act as temporal confounders, influencing engagement or outcomes. The inclusion criteria included: (i) chronic aphasia (> 6 months postonset) resulting from a single unilateral left hemisphere stroke, (ii) aged 18 or above, (iii) spoke fluent Cantonese before stroke, (iv) exhibited word‐finding difficulties, and (v)willing to sign the consent. Conversely, the exclusion criteria included (i) comorbid neurological conditions, (ii) moderate and severe apraxia of speech or dysarthria, and (iii) mental symptoms and obvious emotional agitation. Two baseline measurements, an immediate posttreatment evaluation, and a one‐month follow‐up test were arranged, in which standardized assessments were administered, ranging from word‐level to discourse‐level evaluations. Specifically, linguistic measurements were conducted twice before and after each treatment phase, whereas quality of life outcome measures were collected only at the second baseline and at posttreatment. The assessment arrangement was identical between the two treatment conditions. Figure 1 illustrates a participant who first participated in the ICAP phase, followed by c‐SLT.

### 2.2. Participants

Twelve right‐handed adults with chronic aphasia were recruited for this crossover treatment study, receiving a designated 78 h of treatment. The two treatment phases were conducted alternately, with half of the participants receiving the ICAP first. Although the same group of patients participated in both treatment models, a minimum 6‐month washout period was used, resulting in differences in mean age and time postonset. Table 1 reports the participants′ characteristics and basic information about their communication partners, using the DESCRIBE checklist [20], which was collected during an intake interview at first baseline assessment.

**Table 1:** Demographic information of participants using DESCRIBE (Wallace et al., 2023).

Participants with aphasia	ICAP group	c‐SLT group
Total no. of participants	12	12
Sex (no. of participants)	Male: 7; female: 5	Male:7; female: 5
Age	Mean: 62.08	Mean: 62.17
	Standard deviation: 7.50	Standard deviation: 7.81
	Range: 47–77	Range: 46–76

Comparing the age of the two groups using Mann–Whitney *U* test	*Z* = −0.09, *p* = 0.93	

Education (in years)	Mean: 10.75	Mean: 10.75
	Standard deviation: 3.62	Standard deviation: 3.62
	Range: 6–16	Range: 6–16

Time postonset (in months)	Mean: 40.00	Mean: 40.08
	Standard deviation: 33.82	Standard deviation: 35.31
	Range: 18–140	Range: 12–146

Comparing the time postonset of the two groups using Mann–Whitney‐U test	*Z* = −0.12, *p* = 0.91	

Aphasia types (no. of participants)	Anomia (3); Broca (4); conduction (1); global (1); transcortical motor (3); transcortical sensory (0); Wernicke (0)	Anomia (3); Broca (4); conduction (1); global (1); transcortical motor (3); transcortical sensory (0); Wernicke (0)

Aphasia severity^+^ (no. of participants)	Mild (0); moderate (8); severe (4)	Mild (0); moderate (9); severe (3)

Language of treatment/testing	Cantonese	Cantonese

Primary language (no. of participants)	Spoken language:	Spoken language:
	Cantonese (12)	Cantonese (12)
	Written language:	Written language:
	Chinese (12)	Chinese (12)

Language used (no. of participants)	Spoken language:	Spoken language:
	Cantonese (12)	Cantonese (12)
	English (7)	English (7)
	Written language:	Written language:
	Chinese (12)	Chinese (12)
	English (7)	English (7)

History of condition(s) known to impact communication/cognition	None	None

History of previous stroke	None	None

Lesion hemisphere (no. of participants)	Left (12)	Left (12)

Conditions arising from neurological event (no. of participants)	Apraxia of speech (4)	Apraxia of speech (4)
	Dysarthria (4)	Dysarthria (4)
	Hemiplegia (3)	Hemiplegia (3)
	Hemiparesis (1)	Hemiparesis (1)

Previous exp. of intensive aphasia therapy	0	0

Previous exp. of speech and language therapy	12	12

Previous exp. of app‐based aphasia therapy	0	0

Previous exp. of group aphasia therapy	1	1

Communication partners	ICAP group	c‐SLT group
Total no. of participants	14	12
Sex (no. of participants)	Male: 3; female: 11	Male: 2; female: 10

Age	Mean: 52.21	Mean: 55.00
	Standard deviation: 13.81	Standard deviation: 13.26
	Range: 29–70	Range: 29–71

Relationship to person with aphasia (no. of participants)	Spouse/partners (7); mother/father (0); son/daughter (4); sibling (3); friend/associate (0)	Spouse/partners (6); mother/father (0); son/daughter (3); sibling (3); friend/associate (0)

*Note:* ^+^Based on the scoring methodology of Western Aphasia Battery Revised (WAB‐R; Kertesz, 2007) to classify the severity of aphasia. AQ scores 0–25 are very severe, 26–50 are severe, 51–75 are moderate, and 76 or above are mild.

During the ICAP phase, some participants were accompanied by more than one communication partners, resulting in a larger number than the persons with aphasia. However, a communication partner was mainly involved during the assessment stages of the c‐SLT phase; only the information of the person responsible for the questionnaire was reported. The aphasia quotient of the Cantonese version of the Western Aphasia Battery (CAB) was used to diagnose aphasia type and to categorize severity, in accordance with the scoring methodology of the Western Aphasia Battery Revised (WAB‐R; Kertesz, 2007). The four severity levels identified were mild (AQ ≥ 76), moderate (51–75), severe (26–50), and very severe (0–25).

### 2.3. Primary Outcome Measures

Two comprehensive language functioning tests were selected. The first was the CAB [21], which aimed to assess the participant′s general language abilities and classify aphasia type and severity. Secondly, a more up‐to‐date standardized language test, the Cantonese version of the Comprehensive Aphasia Test (Cant‐CAT) [22], specifically the comprehension and expression parts, was administered to provide a more comprehensive array of the participants′ language abilities. The comprehension tests in the Cant‐CAT tapped different aspects of receptive abilities, and the expressive language tasks included repeating words, sentences, and digits, as well as naming nouns and actions. All these tasks were complementary to those in the CAB.

### 2.4. Secondary Outcome Measures

Two individual language modality tests, the 30‐item Cantonese version of the Boston Naming Test (Cant‐BNT‐30) [23] and the Main Concept Analysis in Cantonese (MCA) [24], were used to assess the participants′ naming and discourse abilities, respectively. The Cant‐BNT‐30 assessed not only naming accuracy but also the scaffolding needed to facilitate successful word retrieval, naming efficiency, and error analysis. The MCA measured the propositions of four sequential picture spoken discourses in terms of accuracy and completeness. As Richardson and Dalton [25] suggested, discourse analysis could predict real‐life communication, and Kong and Wong [26] demonstrated that the total scores of main concept measurements strongly correlated with naive listeners′ perceptions of the clarity and completeness of spoken discourse. Therefore, the use of MCA could provide valuable insights into participants′ out‐of‐clinic communications. In addition, two quality of life outcome measures were included in this study to evaluate how the treatment would affect psychological functioning and communication in daily activities and participation. The Communication Confidence Rating Scale for Aphasia (CCRSA) [27], a patient‐reported outcome measure, was used to assess changes in communication confidence before and after treatment. The Communicative Effectiveness Index (CETI) [28], a communication partner/caregiver‐reported outcome measure, was used to evaluate the aphasia patient′s communication skills in daily contexts.

### 2.5. The ICAP Treatment Condition

**Figure 2:**
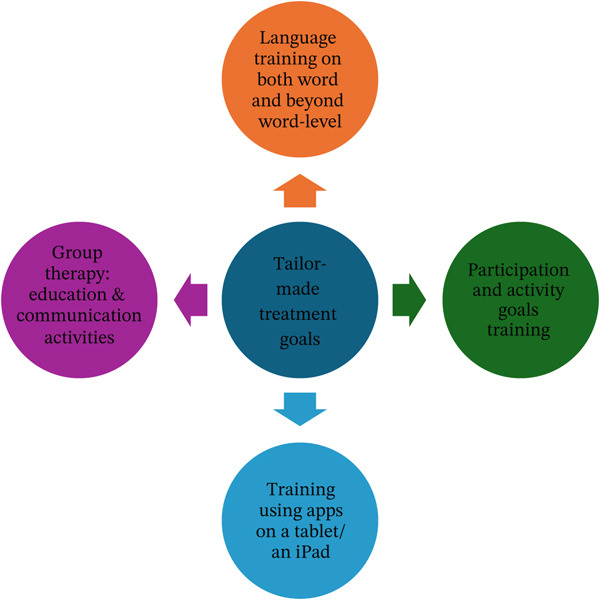
The treatment components of the ICAP in this study.

In the ICAP phase, participants received a total of 39 h of treatment, delivered over 2.5 weeks at an intensity of 3 h/day, 5 days/week. The treatment protocol replicated the program protocol outlined by Wong et al. [10], focusing on both word and beyond‐word levels, and implemented evidence‐based treatment approaches. A short interview with the participant (accompanied by the communication partner/caregiver) was conducted at the first baseline to collect information about hobbies, frequently engaged activities, or upcoming social events, for the purpose of goal setting. In terms of treatment comprehensiveness, participants received 12 h of impairment‐based, 12 h of participation‐based, 9 h of technology‐based, and 6 h of group therapy. Figure 2 outlines the treatment components of an ICAP. All treatments were delivered by either student speech therapists under the supervision of clinical educators or by speech therapists. Supporting Information 2 provides details of the treatment received by participants, along with their baseline aphasia type and severity.

### 2.6. The c‐SLT Condition

Brady et al. [13] acknowledged that “what is considered conventional in one context may not be directly comparable to conventional speech and language therapy in another” (p.142). We adopted the term “conventional” for this treatment condition primarily based on the characteristics of aphasia service delivery in Hong Kong, to contrast with an ICAP treatment model. As the usual care arrangement was highly varied and scattered, dominated by a short treatment hour per therapy session (i.e., around 30 min to less than 2 h), we adopted the treatment frequency observed in university or private clinics to enhance the practical feasibility of this therapy condition. Hence, the c‐SLT phase of this study comprised 39 h of treatment, delivered at an intensity of 2 h per week, once per week, over approximately 5 months. This treatment condition followed a typical local SLT arrangement, consisting of individual language‐only therapy (i.e., impairment‐based) targeting both word and beyond‐word levels. As with the ICAP treatment arrangement, a short interview was conducted before treatment to set goals. Evidence‐based treatment approaches were used, and Supporting Information 3 provides details of the treatments received by each participant. All treatment sessions were delivered by speech therapists.

### 2.7. Interview

All participants, accompanied by communication partners, were interviewed after the completion of both treatment conditions at the posttreatment test of the second phase of treatment. All interviews were conducted by nontreatment clinicians face‐to‐face at the rehabilitation centers, and the interviewees fully understood that the interviewers were collecting research data. The interviewers made field notes and audio recordings during the interviews. The recordings were then transcribed and sent to the participants and communication partners for verification, comment, or correction. Then, the transcriptions were coded and summarized into themes according to the guidelines of Graneheim and Lundman [29].

### 2.8. Statistical Analysis

The two baselines of each treatment model were compared. The stable baselines observed in both models allowed us to rule out spontaneous recovery. We then averaged the two baselines to obtain the pretreatment scores for further analysis.

For individual participant data analysis, the therapeutic effects of each linguistic outcome measure were calculated using the minimum detectable change_90_ (MDC_90_) [30]. Since the quality of life outcome measures had not yet been validated in Cantonese, only the percentage change was reported.

For group‐level analysis, although the data were assessed for normality and passed the normality test, the relatively small sample size (*n* = 12) in each treatment condition may limit the robustness of parametric tests. To ensure the validity and reliability of the statistical analysis under these conditions, nonparametric tests were used to assess the two treatment models. The Wilcoxon signed‐rank test was used to identify immediate treatment effects and maintenance effects, with effect sizes calculated using the rank‐biserial correlation (*r*). Bonferroni correction was applied to all linguistic measures with a significance threshold of *p* < 0.025. To compare the results across the two treatment conditions, linear mixed modeling (LMM) was used. LMM accounted for correlated observations and allowed adjustments for confounders. Specifically, the fixed effects included the two treatment conditions of this study, time of assessment, and the interaction between treatment condition and time; the random effects were participant, and the covariance was baseline aphasia severity and each test′s prescores. Bonferroni correction was applied and the alpha level was adjusted to *p* < 0.025 to account for multiple comparisons. The magnitude of the treatment effect was measured using Cohen′s *d*.

### 2.9. Reliability and Fidelity Measures

In both treatment models, 25% (3/12) of the scoring forms for each test were randomly selected and blindly scored by a nontreatment clinician to measure point‐to‐point interrater reliability. The point‐to‐point measurement used the formula: total agreements/(total agreements + total disagreements) × 100%. The interrater reliability and agreement were excellent (> 95%) for all measures.

Attendance and treatment delivery for each session were charted and confirmed by supervisors. During the ICAP phase, 75% (9/12) completed the entire 39 h of treatment; one participant missed one and a half hours due to lateness, and two participants missed one session (i.e., 3 h) owing to personal arrangements. During the c‐SLT phase, eight treatment schedule rearrangements were noted, with only one session (2 h) failing to arrange a makeup session within the same week. Therefore, the overall completion rate for the c‐SLT phase was 92% (11/12), with only one participant completing 37 h.

## 3. Results

### 3.1. Individual‐Level Analysis

Therapeutic gains or losses were determined using the MDC_90_ for both treatment models at the posttreatment assessment. Table 2 lists the criteria for all primary and secondary measures, along with the total number of gains, nonrespondents, and losses. The total number of gains across measures in the ICAP group ranged from 42% to 75%, while no losses were recorded. In the c‐SLT group, over 90% of participants fell into the “nonrespondent” category across all tests, except for one secondary measure, the error reduction in the Cant‐BNT‐30, where around 83% (10/12) reached the gain threshold.

**Table 2:** Therapeutic gains or losses under the minimum detectable change_90_ criteria for the ICAP and c‐SLT groups.

	CAB		Cant‐CAT				Cant‐BNT‐30				MCA	
	AQ		Comprehension		Expression		Accuracy		Error reduction		Total scores	
	ICAP	c‐SLT	ICAP	c‐SLT	ICAP	c‐SLT	ICAP	c‐SLT	ICAP	c‐SLT	ICAP	c‐SLT
Pre and Posttreatment	7.43	5.63	5.90	3.56	10.03	10.89	3.51	4.03	1.08	0.80	6.37	6.31
Total no. of gain	7	0	5	1	8	0	6	1	7	10	9	1
Total no. of nonrespondents	5	12	7	11	4	12	6	11	5	2	3	11
Total no. of loss	0	0	0	0	0	0	0	0	0	0	0	0

Abbreviations: CAB, the Cantonese version of the Western Aphasia Battery; Cant‐BNT‐30, the 30‐item Cantonese version of the Boston Naming Test; Cant‐CAT, the Cantonese version of the Comprehensive Aphasia Test; CCRSA, the Communication Confidence Rating Scale for Aphasia; CETI, the Communicative Effectiveness Index; MCA, the Main Concept Analysis in Cantonese.

For quality of life outcome measures, all participants in the ICAP group reported improved communication confidence, with increases ranging from 4% to 83%; 58% (7/12) reported increases of more than 20%. Meanwhile, all participants (12/12) in the c‐SLT group also reported increased communication confidence, but the increases tended to be more modest, ranging from 3% to 20%, with only 17% (2/12) reporting increases of more than 10%. The posttreatment communication effectiveness of the ICAP group ranged from 22% to 63%, with 58% (7/12) of communication partners reporting increases of more than 30%. A more subtle change was observed in the c‐SLT group (i.e., 0%–24%); only two communication partners reported increases of more than 10%.

### 3.2. Group‐Level Analysis

**Figure 3:**
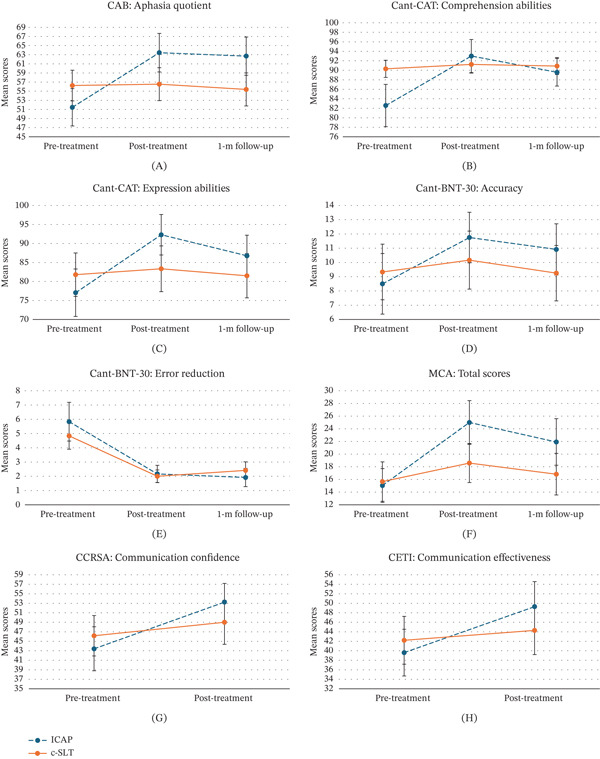
The performance of the two treatment models at pretreatment, posttreatment, and one‐month follow‐up. *Note:* The solid line represents conventional speech and language therapy (c‐SLT), and the dotted line represents the intensive and comprehensive aphasia program (ICAP). Figure 3A–H presents the results at different timepoints. Abbreviations: CAB, the Cantonese version of the Western Aphasia Battery; Cant‐BNT‐30, the 30‐item Cantonese version of the Boston Naming Test; Cant‐CAT, the Cantonese version of the Comprehensive Aphasia Test; CCRSA, the Communication Confidence Rating Scale for Aphasia; CETI, the Communicative Effectiveness Index; MCA, the Main Concept Analysis in Cantonese.

Figure 3a–h illustrates the performance of the two treatment models at pretreatment, posttreatment, and one‐month follow‐up. Across all primary outcome measures (i.e., CAB aphasia quotient, Cant‐CAT comprehension and expression scores), the ICAP group showed substantial increases from pretreatment to posttreatment, followed by gradual decreases at one‐month follow‐up. In contrast, performance changes in the c‐SLT group were more subtle across these standardized assessments. Regarding secondary outcome measures, both the ICAP and c‐SLT groups demonstrated a steep decline in errors, as reflected in the Cant‐BNT‐30, with comparable results observed at posttreatment. At one‐month follow‐up, the c‐SLT showed a very mild bounce‐back, whereas the ICAP group showed a further reduction in errors. Both the ICAP group and the c‐SLT group demonstrated an upward trend in the discourse task, participant‐rated communication confidence, and communication partner/caregiver‐rated communication effectiveness at posttreatment, with larger increases observed in the ICAP group. Detailed descriptive statistics of the two groups are provided in Supporting Information 4.

### 3.3. Primary Outcome Measures

Table 3 presents the group‐level results, in which the two treatment models were analyzed independently. The ICAP group demonstrated significant improvement in the CAB aphasia quotient (*Z* = −3.06, *p* = 0.002, *r* = 0.96), the Cant‐CAT comprehension scores (*Z* = −2.76, *p* = 0.006, *r* = 0.86) and expression scores (*Z* = −3.07, *p* = 0.002, *r* = 0.97) at posttreatment, with large effect sizes. On the other hand, the c‐SLT group showed no statistically significant increases across all primary outcome measures. At one‐month follow‐up, the ICAP group sustained gains in the CAB aphasia quotient and the Cant‐CAT comprehension scores; however, performance on the Cant‐CAT expression scores declined substantially from posttreatment (*Z* = 2.9, *p* = 0.004, *r* = 0.92). Meanwhile, the c‐SLT group also recorded a significant decrease in the Cant‐CAT expression scores (*Z* = 2.63, *p* = 0.009, *r* = 0.83) after the one‐month no‐treatment period.

**Table 3:** Performance comparisons over assessment timepoints with the effect sizes.

	ICAP				c‐SLT			
	Pre and posttreatment comparison		Posttreatment and one‐month follow‐up comparison		Pre and posttreatment comparison		Posttreatment and one‐month follow‐up comparison	
	Significance	Effect size	Significance	Effect size	Significance	Effect size	Significance	Effect size
CAB: Aphasia quotient	*Z* = −3.06^∗^	*r* = 0.96	*Z* = 1.45		*Z* = −0.53		*Z* = 2.14	
Cant‐CAT: Comprehension scores	Z = −2.76^∗^	*r* = 0.86	*Z* = 2.14		*Z* = −1.57		*Z* = 0.82	
Cant‐CAT: Expression scores	Z = −3.07^∗^	*r* = 0.97	*Z* = 2.91^∗^	*r* = 0.92	*Z* = −2.16		*Z* = 2.63^∗^	*r* = 0.83
Cant‐BNT‐30: Accuracy	*Z* = −2.55^∗^	*r* = 0.80	*Z* = 1.25		*Z* = −2.26		*Z* = 2.41^∗^	*r* = 0.76
Cant‐BNT‐30: Errors	*Z* = −2.43^∗^	*r* = 0.76	*Z* = 0.47		*Z* = −2.81^∗^	*r* = 0.89	*Z* = 1.67	
MCA: Total scores	*Z* = −3.06^∗^	*r* = 0.97	*Z* = 2.68^∗^	*r* = 0.85	*Z* = −3.08^∗^	*r* = 0.97	*Z* = 2.36^∗^	*r* = 0.74
CCRSA^+^	*Z* = −3.05^∗^	*r* = 0.97			*Z* = −2.69^∗^	*r* = 0.85		
CETI^+^	*Z* = −3.05^∗^	*r* = 0.96			*Z* = −2.81			

*Note:* (1) Bonferroni correction was applied to all linguistic measures:  ^∗^ *p* < 0.025;  ^∗∗^ *p* < 0.001. (2) +: The quality of life outcome measures adhere to a significance threshold of  ^∗^ *p* < 0.05;  ^∗∗^ *p* < 0.001. (3) Rank‐Biserial Correlation (r): *r* < 0.1 indicates a small effect size; 0.1 ≤ *r* < 0.3 indicates a medium effect size; *r* ≥ 0.3 indicates a large effect size.

Abbreviations: CAB, the Cantonese version of the Western Aphasia Battery; Cant‐BNT‐30, the 30‐item Cantonese version of the Boston Naming Test; Cant‐CAT, the Cantonese version of the Comprehensive Aphasia Test; CCRSA, the Communication Confidence Rating Scale for Aphasia; CETI, the Communicative Effectiveness Index; MCA, the Main Concept Analysis in Cantonese.

### 3.4. Secondary Outcome Measures

Refer to Table 3. The ICAP group revealed significant improvements across all secondary outcome measures at the post‐treatment assessment. These changes indicated large effect sizes (i.e., *r* ranged from 0.76 to 0.97). The c‐SLT group also achieved significant error reduction in the Cant‐BNT‐30 (*Z* = −2.81, *p* = 0.005, *r* = 0.89), the total scores of the MCA (*Z* = ‐3.08, *p* = 0.002, *r* = 0.97), and communication confidence (*Z* = −2.69, *p* = 0.007, *r* = 0.85), indicating large effect sizes. At the one‐month follow‐up, both groups sustained the treatment effect in error reduction as reflected in the Cant‐BNT‐30; however, neither group maintained the improvements in the discourse measure, exhibiting a decline in the total scores of the MCA (i.e., *Z*_ICAP_ = 2.68, *p* = 0.007, *r* = 0.85; *Z*_c−SLT_ = 2.36, *p* = 0.018, *r* = 0.74). Regarding Cant‐BNT‐30 naming accuracy, whereas the ICAP group maintained treatment gains, the c‐SLT group showed a significant decrease from posttreatment levels (*Z* = 2.41, *p* = 0.016, *r* = 0.76).

### 3.5. The ICAP Group Versus the c‐SLT Group

LMM was used to compare the two treatment models at posttreatment and at one‐month follow‐up (see Table 4).

**Table 4:** Comparing the effects of ICAP versus c‐SLT using linear mixed modelling.

	Posttreatment					One‐month follow‐up				
	Type III tests of fixed effects	Standard error	95% CI (lower value, upper value)	*t*‐score	Effect size (Cohen′s *d*)	Type III tests of fixed effects	Standard error	95% CI (lower value, upper value)	*t-*score	Effect size (Cohen′s *d*)
CAB: Aphasia quotient	*F* (1, 56.1) = 37.28, *p* < 0.001	1.73	7.11, 14.06	6.11	2.56	*F* (1, 56.1) = 42.72, *p* < 0.001	1.73	7.86, 14.80	6.54	2.75
Cant‐CAT: Comprehension scores	*F* (1, 57.2) = 4.33, *p* = 0.042	2.69	0.21, 10.97	2.08	—	*F* (1, 57.2) = 0.80, *p* = 0.376	2.69	−2.98, 7.78	0.89	—
Cant‐CAT: Expression scores	*F* (1, 56) = 65.93, *p* < 0.001	1.60	9.81, 16.24	8.12	3.35	*F* (1, 56) = 33.99, *p* < 0.001	1.60	6.14, 12.75	5.83	2.41
Cant‐BNT‐30: Accuracy	*F* (1, 55) = 11.42, *p* = 0.002	0.61	0.84, 3.30	3.38	1.38	*F* (1, 55) = 12.39, *p* = 0.001	0.61	0.93, 3.38	3.52	1.44
Cant‐BNT‐30: Errors	*F* (1, 54.7) = 0.34, *p* = 0.575	0.76	−1.95, 1.09	−0.58	—	*F* (1, 54.7) = 1.49, *p* = 0.227	0.76	−2.45, 0.59	−1.22	—
MCA: Total scores	*F* (1, 54.8) = 28.09, *p* < 0.001	1.33	4.38, 9.68	5.30	2.16	*F* (1, 54.8) = 18.40, *p* < 0.001	1.33	3.03, 8.35	4.29	1.75
CCRSA	*F* (1, 33.2) = 19.45, *p* < 0.001	1.33	3.16, 8.57	4.41	1.81					
CETI	*F* (1, 33.3) = 40.83, *p* = 0.008	1.18	5.15, 9.95	6.39	2.64					

*Note:* (1) Fixed effects included: group, time, group*time. Random effects included: participants. Covariates included: severity, order of treatment and pretreatment scores. (2) Significance is at*p* < 0.025after Bonferroni correction for all tests, except for CCRSA and CETI, where significance is at *p* < 0.025.

Abbreviations: CAB, the Cantonese Version of the Western Aphasia Battery; Cant‐BNT‐30, the 30‐item Cantonese version of the Boston Naming Test; Cant‐CAT, the Cantonese version of the Comprehensive Aphasia Test; CCRSA, the Communication Confidence Rating Scale for Aphasia; CETI, the Communicative Effectiveness Index; MCA, the Main Concept Analysis in Cantonese.

At posttreatment, significant benefits in linguistic performance were observed in the ICAP group compared with the c‐SLT group. These included the CAB aphasia quotient (*d* = 2.56), the Cant‐CAT expression scores (*d* = 3.35), the Cant‐BNT‐30 naming accuracy (*d* = 1.38), and the MCA total scores (*d* = 2.16); all indicated large effect sizes. However, the Cant‐CAT comprehension scores and the Cant‐BNT‐30 error reduction did not show statistically significant differences between the two treatment conditions. Quality of life measures were assessed only at the posttreatment evaluation. The results showed that the ICAP group demonstrated greater communication confidence (*d* = 1.81) and greater communication effectiveness (*d* = 2.64), both with large effect sizes, compared with the c‐SLT group.

At one‐month follow‐up, the ICAP group significantly outperformed the c‐SLT group on most linguistic measures, including the CAB aphasia quotient (*d* = 2.75), the Cant‐CAT expression scores (*d* = 2.41), the Cant‐BNT‐30 naming accuracy (*d* = 1.44), and the MCA total scores (*d* = 1.75); all effects were large.

### 3.6. Structured Interview

All participants (12/12) responded to the three questions. Each interview lasted 10–15 min. For those participants with more severe aphasia, the communication partners joined in and provided multiple options upon communication breakdowns. It was reported that the participants would nod or say “yes” to indicate agreement and would try to use the provided pens and paper, as well as gestures, to convey ideas when disagreeing. Supporting Information 5 provides details on the themes, codes, and example quotes from the interview.

The first question was “How does the ICAP experience differ when compared to the c‐SLT?” Two major themes were summarized: “A new experience of aphasia treatment” and “Sharing of the benefits”. More than half of the participants mentioned the intensity, the duration of training, and some described “like schooling” and “Monday to Friday, just like going to work”. They also highlighted that the design of an ICAP was new to them and allowed them to try different types of therapy. Last but not least, most of them mentioned they felt tired, especially in the first few days, but managed to adapt later. Participants also shared the benefits when responding to this question. Most of the benefits mentioned were related to improved abilities, confidence, and compliments from family, friends, and people in their social circle. Some also mentioned they enjoyed the connections and time in groups.

The second question was “Is there anything you like about the c‐SLT over ICAP?” Three major themes were concluded: “Prolonged interaction”, “Flexibility”, and “Less fatigue”. The participants reported feeling “good,” “less uncertain,” and “secure” after being scheduled for a regular weekly follow‐up for a longer period (i.e., c‐SLT lasted 4 months). They also mentioned the relationship built with the clinician, as “like seeing a friend” and “have more to catch up and share”. Regarding flexibility, most comments concentrated on a c‐SLT being easily fit into their schedule. Some of them also highlighted that they “feel less stressed” or “worried that I missed the training” when on sick leave or for other unchangeable commitments, as it was easier to reschedule than with an ICAP.

The third question was “Is there anything you like about an ICAP over c‐SLT?” In response, three themes emerged: “Achievement,” “Diversified components,” and “Personal goals”. Most participants reported improvements, such as “more talk, talking,” “guess less,” “I tried more to say,” and “easier to think words,” which they or their family and friends had noticed. Additionally, some participants noted that it was more efficient and effective for them to memorize skills or recall learned strategies. Participants also expressed appreciation for experiencing and trying different types of therapy, describing an ICAP as “hard but less boring,” “tired but a lot to learn,” and “group (therapy) is fun,” and for the opportunity to interact with other PWA. Last but not least, some participants mentioned that the high‐intensity, high‐frequency design of an ICAP aligned with their desire to practice more.

## 4. Discussion

### 4.1. The Effects of the Two Treatment Models

The results from the ICAP phase aligned with effects on linguistic domains and quality of life outcomes, as documented in the literature [7, 8], at both the individual and group levels. At the individual level, all participants (12/12) from the ICAP treatment condition achieved at least one therapeutic gain on at least one linguistic measure, and 92% (11/12) achieved at least two therapeutic gains. At the group level, the ICAP condition showed significant improvement across all primary and secondary outcome measures, and most treatment effects were sustained at follow‐up. In the c‐SLT treatment condition, 83% (10/12) of participants achieved therapeutic gains across linguistic measures on an individual basis, with naming errors significantly reduced and sustained the effect at follow‐up on a group basis. The results were consistent with previous findings that SLT could benefit PWA [31].

### 4.2. Comparing the Two Treatment Models

Linguistically, our linear mixed models indicated that the ICAP treatment condition achieved gains significantly greater than the c‐SLT condition at both post treatment and one‐month follow‐up. As we emphasized both word‐level and beyond‐word‐level treatment in both treatment conditions, the greater language gains observed in the ICAP group than in the c‐SLT group might largely be attributed to the intensity of treatment, consistent with the mass practice principle [32]. Interestingly, though most participants (11/12) did not receive direct receptive language treatment in either treatment phase, only the ICAP condition showed significant positive changes at posttreatment and at one‐month follow‐up. This might be attributed to the need to follow instructions, understand questions, and comprehend scenarios during expressive language training, where intensive treatment (i.e., massive practice within a short period of time) further promoted neuroplasticity [13].

Regarding quality of life outcomes, although the two standardized measures used were not validated in our population, they were translated into Chinese and cross‐checked by the authors. The ICAP phase significantly outperformed the c‐SLT phase in CCRSA communication confidence and CETI communication effectiveness. As all participants reported increased communication confidence, this suggests that PWA might feel more confident in communication after receiving therapy, with the roles of treatment intensity and frequency appearing less influential. Considering communication effectiveness, some communication partners/caregivers reported no change or only a very subtle change after the c‐SLT phase, whereas all indicated improved communication effectiveness after the ICAP phase. However, we agreed with Worrall et al. [33] that self‐reported confidence is prone to bias because participants had invested time and effort in the treatments; thus, the result should be interpreted cautiously. This also applied to the communication partner/caregiver‐rated communication effectiveness, as proxies were required to attend group therapy sessions in the ICAP condition, whereas attending treatment sessions in the c‐SLT condition was welcome but not compulsory.

### 4.3. Interview

When the participants were asked to compare the two treatment models, they mainly shared their experiences of joining a new treatment model (i.e., the ICAP), including its intensity and components—they reported fatigue, especially at the very beginning, but stamina increased with time—which was similar to the report by Schütz et al. [34]. They also mentioned the benefits they perceived, including acquired skills, improved communication and confidence, and compliments from their social circles, which were among the treatment model′s unique highlights.

Regarding aspects the participants favored about the c‐SLT over the ICAP, in addition to being less exhausted, they valued the prolonged interaction with the clinician and the flexibility to reschedule. The participants reported psychological benefits of feeling “more stable” and “less uncertain” because their language abilities were monitored weekly by a clinician. Nevertheless, some participants valued the flexibility to reschedule during the c‐SLT condition as “less stressful,” as they did not have to miss any therapy hours. In this study, only one session (out of eight reschedule requests) of the c‐SLT group had not been rearranged, resulting in more actual treatment doses delivered than the ICAP group. The tightly scheduled daily training and the “a cohort begins and ends together” [3] make rescheduling less feasible, resulting in reduced actual dosage delivery, which might also be a confounding factor for the benefits of intensive treatments other than higher dropout rate [1].

When participants mentioned what they liked about the ICAP compared to the c‐SLT, most of their comments focused on the ICAP′s effectiveness in language and communication skills, as well as on group therapy being fun and helping them connect with others. These findings were consistent with those of Babbitt et al. [35] It was noted that some perceived effects (e.g., recalling strategies more efficiently, making more attempts to convey messages in conversations) were not captured in the quantitative measures; our finding further underscores the importance of collecting qualitative data to analyze social interactions and relationships [36]. In addition, their opinions indicated that the diversified treatment components of an ICAP were not just “enjoyment and contentment” [34], but also a sense of self‐recognition, recognizing their efforts and learning.

### 4.4. Limitations and Future Directions

The intensity and frequency of the c‐SLT treatment model reflected the frequency of aphasia services offered in university or private clinics [18]. Therefore, these findings should be interpreted with caution, as they do not fully represent local care for aphasia, where therapy arrangements are highly variable and often provide services with lower intensity and frequency than those in university or private clinics.

The participant pool for this experimental design was small, and most participants had expressive aphasia with severity levels ranging from moderate to severe, which might have limited the generalizability of the results to the broader aphasia community. Moreover, a small sample size is often associated with greater sampling variability and greater susceptibility to outliers, which may distort the validity of the resulting effect sizes. This study explored potential differences and benefits between the treatment models; however, caution is warranted owing to the small sample size.

The interview responses provided additional information about changes in daily participation that the quantitative assessment (i.e., CETI) did not capture, such as activities that a participant had not previously engaged in but began participating in after the treatment. This demonstrates the CETI′s limited evaluations of how a treatment model affected one′s daily activities and participation. Future studies could ask participants and their proxies to list the activities (if any) in follow‐up interviews. Nevertheless, the interview only sought feedback on how participants experienced the two treatment models; in‐depth investigations into each component of the ICAP model or comparisons of the c‐SLT model with previous aphasia therapy experiences, satisfaction levels, and communication partners/caregivers′ feedback, were not conducted. Further investigation into stakeholders′ perspectives on an ICAP, with respect to the WHO‐ICF domains, would help identify which treatment components matter most and may inform future designs, such as the designated treatment time or the proportions of each component.

## 5. Conclusions

Our findings indicated that both the ICAP and the c‐SLT models could benefit the linguistic abilities and quality of life outcomes of PWA at the chronic stage. Subsequently, the ICAP model has been identified as exhibiting stronger acquisition and maintenance effects than the c‐SLT model, including overall language functioning, discourse abilities, psychosocial aspects, and participation in out‐of‐clinic environments. This evidence could serve as a foundation for introducing and implementing an ICAP, a novel treatment model, in local aphasia care.

The interviews with participants underscored the psychological benefits and flexibility of a c‐SLT approach compared to the ICAP model. On the other hand, the perceived improvements in language and communication skills, increased communication confidence, and the diversified treatment components were notable highlights of the ICAP model, as reflected in participants′ feedback. These findings offer valuable insights from user perspectives that rehabilitation service providers should consider when evaluating eligibility and managing expectations during the recruitment stage.

## Author Contributions

Cherie Wan‐Yin Wong: conceptualization, methodology, investigation, formal analysis, writing—original draft, and project administration. Anthony Pak‐Hin Kong: conceptualization, methodology, review and edit, supervision, resources, and funding acquisition. Ada Wai‐Sze Chu: validation, investigation, and project administration.

## Funding

This work was supported by the Hong Kong SAR Government Research Grants Council, 17620723.

## Ethics Statement

We have obtained human ethics approval from the University′s Human Research Ethics Committee (HREC) for ethical clearance for research involving human participants. The HREC Reference Number is EA230094. Participant recruitment, data collection, and analysis commenced after ethical approval was granted.

## Consent

All participants signed the written consent to participate and to publish.

## Conflicts of Interest

The authors declare no conflicts of interest.

## Supporting information


**Supporting Information** Additional supporting information can be found online in the Supporting Information section. The CARE guideline checklist. Supporting Information 2 The details of treatment received by participants, along with their aphasia severity, type, and comorbidity (if any) of the ICAP treatment condition. Supporting Information 3 The details of treatment received by participants, along with their aphasia severity, type, and comorbidity (if any) of the c‐SLT treatment condition. Supporting Information 4 Descriptive statistics of the ICAP group and c‐SLT group. Supporting Information 5 The themes, codes, and example quotes from the interview.

## Data Availability

The data that support the findings of this study are available from the corresponding author upon reasonable request.
