# Supplementary material for: An Intensive and Comprehensive Aphasia Program Versus a Conventional Speech and Language Therapy: A Dose‐Controlled, Crossover Experimental Study
Source: Stroke Res Treat. 2026 Jun 13;2026:2669488. doi: 10.1155/srat/2669488 (PMC13263642; doi:10.1155/srat/2669488)
Supplement: Supplementary file 1 — Supporting Information Additional supporting information can be found online in the Supporting Information section. The CARE guideline checklist. Supporting Information 2 The details of treatment received by participants, along with their aphasia severity, type, and comorbidity (if any) of the ICAP treatment condition. Supporting Information 3 The details of treatment received by participants, along with their aphasia severity, type, and comorbidity (if any) of the c‐SLT treatment condition. Supporting Information 4 Descriptive statistics of the ICAP group and c‐SLT group. Supporting Information 5 The themes, codes, and example quotes from the interview. [file SRAT-2026-2669488-s001.docx]

Supplementary 2.

The details of treatment received by participants, along with their aphasia severity, type, and comorbidity (if any) of the ICAP treatment condition

|  | **Impairment-based treatment** | | **Participation-based treatment** | **Group therapy** | | | **Tech-based: language apps** |
| --- | --- | --- | --- | --- | --- | --- | --- |
| **Cohort 1** | Word level | Beyond word level |  | Communication activities | CPT | Education about aphasia |  |
| P001  (moderate TM, w/mild dysarthria) | Naming: semantic cueing approach🡪 SFA | 3-element sentence productions | Dialogue script training of ordering food and health check + Role Play+ PACE + Compensatory strategies | Barrier games using PACE  Toaster meeting, Singing  Compensatory strategies |  |  |  |
| P002  (moderate anomia) | Naming: SFA | Extended VNest+ Discourse-story | Passage script of stroke experience + Role Play + Daily planning | Barrier games using PACE  Toaster meeting  reciprocal scaffolding approach |  |  |  |
| **Cohort 2** | | | | | | | |
| P003  (severe global, w/mild AOS) | Naming: semantic cueing approach  Comprehension: Word-picture verification | 2-element sentence production and comprehension | Dialogue script training of ordering food + Role Play + PACE + Compensatory strategies | Barrier games using PAC E | CPT | Education about aphasia | **Tech-based: language apps** |
| P004  (moderate anomia) | Naming: SFA | Extended VNest + Discourse- story | Passage script of hobbies, self-introduction, stroke experience + Activity planning | Barrier games using PACE  Toaster meeting  reciprocal scaffolding approach |  |  |  |
| **Cohort 3**  Group 1 | | | | | | | |
| P006  (moderate TM, w/mild AOS) | Naming: semantic cueing approach🡪 SFA | 2-3-element sentence production | Dialogue script training of hobbies + Role Play + PACE | Barrier games using PACE  Toaster meeting, Singing  Compensatory strategies | CPT | Education about aphasia | **Tech-based: language apps** |
| P008  (moderate anomia) | Naming: SFA | Extended VNest+ Discourse- story | Dialogue script training of ordering food + Passage script of self-introduction, stroke experience | Barrier games using PACE  Toaster meeting  reciprocal scaffolding approach |  |  |  |
| Group 2 | | | | | | | |
| P005  (severe Broca, w/mild AOS) | Naming: semantic cueing approach | 2-element sentence production | Dialogue script training of ordering food and grocery shopping + Role Play + PACE | Barrier games using PACE  Singing | CPT | Education about aphasia | **Tech-based: language apps** |
| P007  (severe Broca) | Naming: semantic cueing approach 🡪 SFA | 2-3-element sentence production | Dialogue script training of ordering food and self-introduction + PACE | Barrier games using PACE  Toaster meeting, Singing  Compensatory strategies |  |  |  |
| **Cohort 4**  Group 1 | | | | | | | |
| P009  (severe TM, w/mild dysarthria) | Naming: GVT | 2-3-element sentence production | Dialogue script training about family + Role Play + PACE | Barrier games using PACE  Compensatory strategies  Singing | CPT | Education about aphasia | **Tech-based: language apps** |
| P011  (moderate Broca, w/mild dysarthria) | Naming: semantic cueing approach SFA | VNest + Extended VNest | Dialogue script training of ordering food + Role Play + Conversational coaching | Barrier games using PACE  Compensatory strategies  Singing |  |  |  |
| Group 2 | | | | | | | |
| P010  (moderate condition) | Naming: SFA | Extended Vnest + Discourse- story | Role Play + PACE + Compensatory strategies on numbers | Barrier games using PACE  Toaster meeting | CPT | Education about aphasia | **Tech-based: language apps** |
| P012  (severe TM, w/mild dysarthria) | Naming: semantic cueing approach | 3-element sentence production | Dialogue script training of self-introduction + Role Play+ PACE | Barrier games using PACE  Toaster meeting |  |  |  |

Supplementary 3. The details of treatment received by participants, along with their aphasia severity, type, and comorbidity (if any) of the c-SLT treatment condition

|  |  | **Impairment-based treatment** | |
| --- | --- | --- | --- |
| Participant | Aphasia severity & type (comorbidity if any) | Word level | Beyond word level |
| P001 | Moderate TM, w/mild dysarthria | Naming: semantic cueing approach | 2-3-element sentence productions |
| P002 | Moderate Anomia | Naming: SFA | Extended VNest |
| P003 | Severe Global | Naming: semantic cueing approach  Comprehension: Word Picture verification | 2-element sentence production and comprehension |
| P004 | Moderate Anomia | Naming: SFA | Discourse- story |
| P005 | Severe Broca, w/mild AOS | Naming: semantic cueing approach | 2-element sentence production |
| P006 | Moderate TM, w/mild AOS | Naming: semantic cueing approach | 2-element sentence production |
| P007 | Moderate Broca | Naming: SFA | 2-3-element sentence production |
| P008 | Moderate Anomia | Naming: SFA | Extended VNest |
| P009 | Severe Broca, w/mild AOS | Naming: GVT | 2-element sentence production |
| P010 | Moderate conduction | Naming: SFA | Discourse-story |
| P011 | Moderate Broca, w/mild dysarthria | Naming: SFA | Extended VNest |
| P012 | Moderate TM, w/mild dysarthria) | Naming: semantic cueing approach | 2-3-element sentence productions |

Supplementary 4.

Descriptive Statistics of the ICAP group and c-SLT group

|  | Mean | | Standard Deviation | | Standard error | | Minimum | | Maximum | |
| --- | --- | --- | --- | --- | --- | --- | --- | --- | --- | --- |
|  | ICAP | c-SLT | ICAP | c-SLT | ICAP | c-SLT | ICAP | c-SLT | ICAP | c-SLT |
| CAB: Aphasia quotient |  |  |  |  |  |  |  |  |  |  |
| Pre-treatment | 51.47 | 56.25 | 14.20 | 11.68 | 4.10 | 3.37 | 29.40 | 31.00 | 74.00 | 72.00 |
| Post-treatment | 63.45 | 56.54 | 14.60 | 12.49 | 4.22 | 3.61 | 39.00 | 30.00 | 83.90 | 75.40 |
| 1-m follow-up | 62.70 | 55.40 | 14.49 | 12.52 | 4.18 | 3.61 | 37.00 | 29.00 | 82.10 | 74.00 |
| Cant-CAT: comprehension scores |  |  |  |  |  |  |  |  |  |  |
| Pre-treatment | 82.58 | 90.31 | 15.41 | 6.21 | 4.45 | 1.79 | 45.00 | 82.50 | 102.75 | 103.50 |
| Post-treatment | 93 | 91.25 | 12.08 | 6.32 | 3.49 | 1.83 | 72.50 | 84.00 | 112.00 | 104.00 |
| 1-m follow-up | 89.58 | 90.90 | 10.04 | 6.22 | 2.90 | 1.80 | 68.00 | 84.00 | 110.00 | 105.00 |
| Cant-CAT: expression scores |  |  |  |  |  |  |  |  |  |  |
| Pre-treatment | 77.04 | 81.79 | 21.66 | 19.84 | 6.25 | 5.73 | 28.00 | 51.00 | 106.50 | 108.00 |
| Post-treatment | 92.29 | 83.33 | 18.41 | 20.82 | 5.32 | 6.01 | 60.00 | 50.00 | 117.00 | 110.00 |
| 1-m follow-up | 86.75 | 81.50 | 18.82 | 20.16 | 5.43 | 5.82 | 54.00 | 50.00 | 111.00 | 110.00 |
| Cant-BNT-30 |  |  |  |  |  |  |  |  |  |  |
| Accuracy: Pre-treatment | 8.50 | 9.33 | 7.37 | 6.76 | 2.13 | 1.95 | 0.00 | 1.00 | 25.00 | 22.00 |
| Accuracy: Post-treatment | 11.75 | 10.17 | 6.14 | 7.04 | 1.77 | 2.03 | 2.00 | 1.00 | 23.00 | 22.00 |
| Accuracy:1-m follow-up | 10.92 | 9.25 | 6.23 | 6.74 | 1.80 | 1.95 | 1.00 | 0.00 | 23.00 | 22.00 |
| Error reduction: Pre-treatment | 5.83 | 4.83 | 4.70 | 3.21 | 1.36 | 0.93 | 0.00 | 0.00 | 14.00 | 12.00 |
| Error reduction: Post-treatment | 2.17 | 2.00 | 2.08 | 1.54 | 0.60 | 0.44 | 0.00 | 0.00 | 7.00 | 5.00 |
| Error reduction:1-m follow-up | 1.92 | 2.42 | 2.23 | 2.07 | 0.65 | 0.60 | 0.00 | 0.00 | 6.00 | 6.00 |
| MCA: Total scores |  |  |  |  |  |  |  |  |  |  |
| Pre-treatment | 15.04 | 15.67 | 9.23 | 10.78 | 2.67 | 3.11 | 0.00 | 0.00 | 30.00 | 37.00 |
| Post-treatment | 25.00 | 18.58 | 11.92 | 10.66 | 3.44 | 3.08 | 4.00 | 2.00 | 39.00 | 38.00 |
| 1-m follow-up | 21.92 | 16.83 | 12.73 | 11.37 | 3.68 | 3.28 | 0.00 | 0.00 | 40.00 | 40.00 |
| CCRSA |  |  |  |  |  |  |  |  |  |  |
| Pre-treatment | 43.42 | 46.17 | 16.03 | 14.69 | 4.63 | 4.24 | 18.00 | 25.00 | 70.50 | 68.00 |
| Post-treatment | 53.25 | 49.00 | 13.64 | 16.03 | 3.94 | 4.63 | 28.00 | 25.00 | 75.00 | 70.00 |
| CETI |  |  |  |  |  |  |  |  |  |  |
| Pre-treatment | 39.62 | 42.22 | 17.05 | 17.42 | 4.92 | 5.03 | 11.10 | 15.00 | 59.00 | 61.00 |
| Post-treatment | 49.30 | 44.29 | 18.31 | 17.67 | 5.28 | 5.10 | 17.50 | 15.00 | 69.40 | 65.00 |

Supplementary 5.

The themes, codes, and example quotes from the interview

| Q1. How does the ICAP experience differ when compared to the c-SLT? | | |
| --- | --- | --- |
| Themes | Codes | Example quotes |
| A new experience of aphasia treatment | Very intensive  Much longer sessions  Monday to Friday, just like going to work  Just like schooling  Training everyday, never tried before  Training with family members  Sophisticated treatment  More tired  Trying different things  Very tired at the beginning and then adapted to it  The total hours of ICAP training exceeded the sum of my previous two years | “…need to nap in the afternoon, haha, (feel) more tired…”  “A lot of things to try, including…. Apps… and talkings in groups”  “The first few days very tired, then I get used to the schedule”  “With her, lesson , with her”  “Good (with gesture thumbs up)… sleep (snoring sound with gestures of sleeping )”  “very new, new to me”  “a lot, lot, (series of gesture)… hahahaha (laugh)…(gesture:thumbs up) good”  “I’ve never done so much training before, in 2 weeks. Many more times than previous”  “I don’t know. Er…er…er… One, two, three, four, three days tired. Then, then, okay, okay, okay (with gestures)” |
| Sharing of the benefits | Talk more  Make new friends  More organized speech  Friends/family members said I have improved in speaking  Friends/family members commented that they could understand more I said  More confidence  Easier for me to say something  Fun in groups  Learn to use the language apps | “Group…mates, we understand (us)”  “Speak better, talk better”  “My friends said I improved obviously, than before”  “My wife said I speak clearly”  “Social worker said she understands me (my expression) more”  “more…more…more… good”  “I talk… talk…talk….try more talk…”  “Know new apps. Apps good (for) naming, I sometimes practice”  “I think, think, improve more, more improve” |
| Q2. Is there anything you like about the c-SLT over ICAP? | | |
| Themes | Codes | Example quotes |
| Prolonged interaction | Training lasts for four months  Built relationships with the clinician  Like meeting friends on a regular basis  Regular follow-up, able to consult the clinician  Feel good to meet the clinician for a longer period of time  More to share and to update the clinician | “… (feel) good to meet, practice, for four months”  “seeing in a week, more to update and share”  “like visiting a friend and chat”  “worry less, …(have) someone to monitor my speech (language abilities)”  “less uncertain of not having therapy”  “Don’t have to think hard (compared to ICAP) an answer to share (with the clinician)” |
| Flexibility | Don’t have to rearrange other activities  Don’t need to worry about missing a training because easier to make-up  Easier to re-arrange time  Less stressful of getting sick  Easily fit in daily schedule | “no need to reschedule other activities”  “…(ICAP), you missed one, three hours, no, no. I lost the training. Hard to find another day”  “easier to make-up, in case suddenly (personal events)”  “Easier to manage (the time)” |
| Less fatigue | Less tired/fatigue | “…(ICAP) I very concentrated, long, very tired. Once a week, easy easier”  “less tired”  “more energy for other activities”  “can go to exercise (physiotherapy) in the afternoon”  “no…no…no… sleep…no…no…nap”  “Piece of cake” |
| Q3. Is there anything you like about ICAP over the c-SLT? | | |
| Themes | Codes | Example quotes |
| Achievement | More improvement  More confidence  Greater progress made  More attempts in  Friends/family members/social workers/church-mates noticed my improvement  Better remembering of what I have learnt  Faster pickup/less time spent on recalling)  I learnt a lot strategies  Remember better the skills | “…talk to others, more talk, talking”  “…said I’m better speak…”  “…said I’m more organized, guess less”  “I don’t have to recall what I learnt last week, oh no, think think think. Waste less time”  “I seems better, better more obviously”  “Easier to think words”  “I tried more to tell, tell her know”  “They said I improve more, speak better than before”  “…practice, good, a lot (gesture: thumbs up)”  “I remembered better the skills, not all, some, some, better, easier” |
| Diversified components | Group is fun  Able to practice with others in groups  A lot to learn, sometimes apps, scripts, sentences, words etc.  Very good to have training everyday  I tried a lot  Hard but not bored  Tired but a lot to learn | “…a lot to learn in a day, sometimes apps, this, that, role play, games, very fun and practical”  “practice…together (in groups), patience, not afraid talk”  “Practice everyday, different tasks. Honestly it is a bit tired but really learn a lot”  “Tried different things in one “course””  “I practice, practice and can show (off) to others (in group therapy)”  “Just like a many courses meal” |
| Personal goals | I want to have more practice  I want to learn more, talk more  I want to find someone to talk, practice daily | “I know it takes time, and need practice, more more, I want to practice more”  “I want to join more”  “I don’t know how to improve, I need someone to teach me and practice with me”  “All my family (members) need to work. I want someone to talk, practice daily”  “…to improve more and (ICAP) is everyday practice” |
